# Analysis of Factors Relevant to the Severity of Symptoms in Children and Adolescents with Postural Orthostatic Tachycardia Syndrome

**DOI:** 10.3390/children12040474

**Published:** 2025-04-07

**Authors:** Yali Cao, Ping Liu, Bo Li, Yingqian Zhang, Junbao Du, Hongfang Jin, Ying Liao

**Affiliations:** 1 Department of Pediatrics, Children’s Medical Center, Peking University First Hospital, Beijing 102627, China; 18900908@hebmu.edu.cn (Y.C.); pingliu@pku.edu.cn (P.L.); drjunbaodu@pku.edu.cn (J.D.); jinhongfang@bjmu.edu.cn (H.J.); 2 Department of Cardiology, Children’ Hospital of Hebei Province, Shijiazhuang 050031, China; 90100103@hebmu.edu.cn; 3 Hebei Provincial Key Laboratory of Pediatric Cardiovascular Disease, Shijiazhuang 050031, China; 90100102@hebmu.edu.cn; 4 State Key Laboratory of Vascular Homeostasis and Remodeling, Peking University, Beijing 100191, China

**Keywords:** postural orthostatic tachycardia syndrome, symptom severity, relevant factor, corrected QT interval dispersion, symptom score, children

## Abstract

**Objectives:** The current study aims to investigate the factors associated with the severity of conditions for pediatric cases with postural orthostatic tachycardia syndrome (POTS). **Methods:** Patients hospitalized and first diagnosed with POTS were retrospectively included and reviewed. The severity of symptoms was evaluated by symptom scores (SSs). Multiple Spearman correlation analyses and multiple linear regression analyses were used to determine factors independently associated with SS. Patients were divided into the mild (SS ≤ P25) and severe (SS ≥ P75) groups to test the distinguishing efficiency of the candidate factors. The efficiency of each independently correlated factor in indicating the condition of children with POTS was assessed by the receiver operating characteristic (ROC) curve. **Results:** A series of 296 pediatric patients aged 5–17 years suffering from POTS were included. Multiple Spearman correlation analyses and multiple linear regression analyses showed that corrected QT interval dispersion (QTcd) was independently correlated with SS (*p* < 0.05). QTcd can be used to suggest the severity of POTS symptoms, and the area under the curve (AUC) was 0.986 (95% CI 0.976–0.997). At a threshold of QTcd = 45 ms, the sensitivity and specificity were, respectively, 94.0% and 91.8% for symptom severity indication. **Conclusions:** In pediatric cases with POTS, QTcd was positively correlated with their symptom severity and exhibited a strong indicative value. A QTcd of 45 ms was a valid cut-off value for indicating symptom severity.

## 1. Introduction

Postural orthostatic tachycardia syndrome (POTS) manifests as symptoms of chronic orthostatic intolerance [1,2], featuring persistent sinus tachycardia during an upright posture [2,3] without significant changes in blood pressure and commonly afflicting children and adolescents. Symptoms of orthostatic intolerance are frequent and recurrent, characterized by dizziness, headache, palpitations, chest tightness, tremors, generalized weakness, blurred vision, pallor, cognitive difficulties, and even syncope in some cases [4,5,6]. In addition, POTS may also present with symptoms unrelated to posture (e.g., malaise, nausea, chronic pain) and systemic symptoms (e.g., fatigue, sleep disturbances, inattention) [4,7], often with a chronic course (≥6 months) [8]. A study on young males found that the prevalence of POTS was nearly 10% [9], while the prevalence in females was even higher, with the ratio of female to male patients being approximately 4–6:1 in one study [10]. A study by Lin et al. involving 600 Chinese children and adolescents in a single center found a prevalence of 6.8% for POTS [11]. As a disorder involving multiple systems, POTS seriously compromises the mental condition of affected children and dramatically reduces their standard of living [12,13,14,15,16]. Due to the diversity of the disease’s manifestations and inconsistencies in its understanding, at present, the knowledge of POTS in children is far from sufficient, which can directly affect the management of the syndrome. Exploring the factors associated with the condition of children with POTS is conducive to improving the understanding of pediatric POTS, helping to screen those with severe conditions for stratified management, and guiding individualized treatment on this basis.

Previously, in studies on symptom severity in pediatric patients affected by POTS, researchers found that plasma homocysteine (Hcy) and sulfur dioxide levels were positively associated with symptom severity caused by elevated heart rate during the upright position [17,18], whereas serum resistin levels were negatively related to both [19]. Although these indicators may indicate the severity of symptoms, the detection of Hcy and resistin requires enzyme-linked immunosorbent assay; on the other hand, sulfur dioxide is detected by high-performance liquid chromatography analysis. These two assays have rather complicated procedures that limit the detection of these indicators at all levels of hospitals, so it is especially crucial to search for simple and easily accessible indicators.

Currently, hypovolemia, abnormal vascular function due to neuropathy and disturbance of autonomic function are the three main recognized mechanisms in POTS [20,21]; therefore, we collected baseline data (involving sex, age, height, weight, and BMI) and those potential indices related to the mechanisms in children with POTS, such as indices related to blood volume [22,23,24,25] [including blood cell count, serum ions, 24 h urinary ion excretion, 24 h urine volume, urine specific gravity (SG)], indicators related to vascular function [26] [including serum uric acid (UA), systolic blood pressure (SBP) and diastolic blood pressure (DBP)] and indicators related to autonomic function [27,28,29,30,31] [including heart rate (HR), maximum QT interval (QTmax), corrected QTmax (QTcmax), minimum QT interval (QTmin), corrected QTmin (QTcmin), corrected QT interval dispersion (QTcd), left ventricular ejection fraction (LVEF), left ventricular fraction shortening (LVFS), and standard deviation of NN intervals (SDNN)]. By retrospectively analyzing these metrics, the current study aimed to explore biomarkers linked to symptom severity and ultimately to improve the effectiveness of the management for children with POTS, as well as the quality of survival of children with POTS.

## 2. Materials and Methods

### 2.1. Patients and Study Design

**Figure 1:**
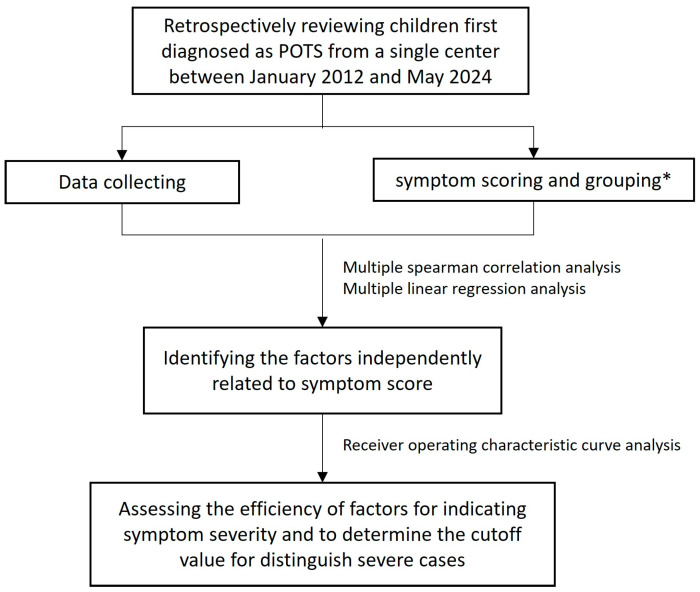
The flow chart of study design. * Patients were divided into the mild (symptom score ≤ the 25th percentile) group and severe (symptom score ≥ the 75th percentile) group.

Individuals from the Pediatric Department at Peking University First Hospital with a definitive diagnosis of POTS participated in the present study. Figure 1 illustrates the flow chart of subject inclusion and study design.

Inclusion criteria: (1) Pediatric patients were first hospitalized and had a diagnosis of POTS between January 2012 and May 2024; (2) 5–17 years old; (3) those with complete data, including history and physical examination records, basic laboratory assays, echocardiography, 12-lead electrocardiogram (ECG), 24 h Holter ECG monitoring, 10 min active standing test and head-up tilt test (HUTT).

Exclusion criteria: (1) girls who were menstruating when they were admitted; (2) individuals who had a history of infections within the two weeks prior to admission; (3) those who received medications that can affect their heart rate or blood pressure when hospitalized or stopped such medications within 5 half-lives prior to admission; (4) those whose electrocardiograms showed non-sinus rhythm; (5) those with comorbidities such as confirmed cardiovascular, cerebral, thyroid, adrenal disorders, hepatic and renal insufficiency and any others with definite systemic diseases; (6) those who had received any treatment for POTS before their admission.

Diagnostic criteria for POTS [16]: (1) recurrent symptoms of orthostatic intolerance, consisting of dizziness, headache, chest stuffiness, cardiopalmus, malaise, tremor, blurred vision, post-exercise discomfort as well as post-orthostatic syncope; (2) symptoms are usually aggravated by susceptibility factors, such as deconditioning, infection, prolonged standing, stuffy environment or emotional stress; (3) the upright heart rate is over-elevated throughout the 10 min active standing test or HUTT observing the following criteria: a normal heart rate can be observed in the supine position but the heart rate can elevate by ≥40 bpm within 10 min after the beginning of standing or tilting, or can achieve a maximum ≥130 bpm in children aged 6 to 12 years, or ≥125 bpm in adolescents aged 13 to 18 years, and accompanied with no orthostatic hypotension (blood pressure decline by <20/10 mmHg); (4) excluding neurological, cardiovascular, or metabolic disorders that may cause the above symptoms.

### 2.2. Ethics

This study obtained approval from the Institutional Ethics Committee of Peking University First Hospital (protocol code 2024-665; approval date: 12 December 2024), and the informed consent was waived by the Institutional Ethics Committee of Peking University First Hospital.

### 2.3. Symptom Scores and Groupings

Symptom scores (SSs) for children with POTS: SSs were calculated according to children’s orthostatic intolerance symptoms and the frequencies of their occurrences during the 1 month prior to admission. The symptoms for evaluation included dizziness, chest stuffiness, headache, palpitations, sweating, nausea, tremors, blurred vision, inattention, and syncope. Symptom frequencies were scored on the following scale [22]: asymptomatic was recorded as 0; once monthly was recorded as 1; twice to 4 times monthly was recorded as 2; twice to 7 times per week was recorded as 3; and appearing more than once daily was recorded as 4. Each symptom’s score was obtained separately by this scale, after which all of the symptoms’ scores were summed to obtain the total SS score.

Groupings of pediatric patients suffering from POTS: The children were grouped using the quartile method. The mild group contained those with SS ≤ the 25th percentile of the SS (P25), while the severe group included those with SS ≥ the 75th percentile of the SS (P75) based on all participants.

### 2.4. Data Collection

We collected all patients’ medical records, including baseline data, detailed medical history, physical examination, and results and diagnostic information of laboratory tests, echocardiography, 12-lead ECG, 24 h Holter, 10 min active standing test, or HUTT, with the Medical Record Digital System (Kaihua, Beijing, China). One researcher collected and recorded the data using Excel software, and another researcher was responsible for checking the data.

Variables collected: (1) baseline data of children with POTS, including sex, age, height, weight, and BMI; (2) SSs of children at admission; (3) indicators related to blood volume, including blood cell counts, serum ions, 24 h urinary ion excretion, 24 h urine volume, urine SG, [among them, blood cell counts included red blood cell count (RBC), hematocrit (HCT), hemoglobin (HGB), mean corpuscular volume (MCV), mean corpuscular hemoglobin (MCH), mean corpuscular hemoglobin concentration (MCHC), red cell distribution width (RDW), platelet count (PLT), mean platelet volume (MPV), platelet hematocrit (PCT) and platelet distribution width (PDW); serum ions including serum sodium, serum potassium, and serum chloride; 24 h urinary ion excretion including 24 h urinary sodium, potassium, and chloride excretion]; (4) indices related to vascular function, including SBP, DBP, and serum UA; and (5) indices related to autonomic function, including HR, QTmax, QTmin, QTcmax, QTcmin, QTcd in the 12-lead ECG, SDNN in the 24 h Holter reports, LVEF, and LVFS.

### 2.5. Laboratory Examinations

The blood and urine specimen testing during the current study was performed by the Laboratory Department in our medical center. Blood cell counts were detected by a complete blood cell analyzer (XN10-B4, SYSMEX, Kobe, Japan), urine routine was detected by a fully automated urinalysis line (UF-5000/UC-3500, SYSMEX, Kobe, Japan), and blood biochemistry and 24 h urine tests were detected by a fully automated biochemistry (AU-5800, BECKMAN, Brea, CA, USA).

### 2.6. Twelve-Lead ECG Tracings, Measurements, and Calculations

ECG examinations were performed using the electrocardiograph (FX-7402, Fukuda Denshi, Tokyo, Japan). Patients were requested to breathe steadily during the examination, lying flat in a quiet, temperature-controlled, and disturbance-free environment. Twelve-lead ECGs were placed by trained examiners according to a standardized procedure, and the results were recorded on the thermal paper of which the voltage was 1 mv/cm and the movement speed was 25 mm/s. The ECGs were qualified as containing the complete lead labeling, the paper speed, and the voltage markers. The graphic baseline of the 12-lead ECG was smooth, and the waveforms were clearly recognizable. The ECG measurements were specified as follows: all measurements were performed in lead II using the PR segment as the isoelectric line, and the QT interval referred to the period between the beginning of the QRS complex and the end of the T wave. RR was the standard ECG RR interval for sinus rhythm. The heart rate was corrected by the Bazett formula to obtain the corrected QTmax (QTcmax, QTcmax = QTmax/RR^1/2^) and the corrected QTmin (QTcmin, QTcmin = QTmin/RR^1/2^). Both QTmax and QTmin were expressed in milliseconds. QTcmax minus QTcmin yielded the corrected QT interval dispersion (QTcd).

### 2.7. Ten-Minute Active Standing Test and HUTT

All patients underwent the 10 min active standing test, and a basic HUTT was performed to assist in the diagnosis if the patient with symptoms of orthostatic intolerance did not fulfill the diagnostic criteria for POTS during the 10 min active standing test or those with syncope.

Protocol for 10 min active standing test [16]: the participant was arranged to lie supine spontaneously in a dimly lit setting with appropriate temperature for at least 10 min, and after the heart rate stabilized, heart rate as well as blood pressure were monitored and separately recorded every 1 min in the supine position. After that, the participant stood up actively for another 10 min, continuing to record heart rate and blood pressure every 1 min. All discomfort of the participant was recorded simultaneously during the test. If the patient experienced extreme discomfort and could not persist, the procedure was immediately terminated, and they were helped to lie down for rest.

Basic HUTT protocol [16,32,33]: The participant discontinued any vasoactive drugs for over 5 half-lives, avoided any foods like tea or coffee that might affect the autonomic state, and fasted for more than 4 h prior to the test. The examination environment was relaxed, undisturbed, with low light levels, and at a suitable temperature, and was scheduled in the morning. HUTT was performed on an inclined bed (SHUT-100A, Jiangsu Stanley Company, Jiangyin, China), and a multilead electrocardiogram monitor (GE, Schenectady, NY, USA) was used to continuously record the heart rate and electrocardiogram. Hemodynamic changes were recorded by a Finapres Medical Systems (FMS) non-invasive continuous blood pressure monitor (FinometerPRO, FMS company, Amsterdam, The Netherlands). The participant was asked to avoid bending their legs and lie quietly for 10–30 min to constantly record their basal heart rate, ECG, and blood pressure. When the above indicators were stabilized, the tilting bed’s angle was adjusted so that the participant was kept in a 60-degree tilt with their head in the high and feet in the low position, and the continuous recordings of the heart rate, blood pressure, and ECG was obtained until a positive response was observed or the procedure was completed in 45 min after tilting. Once a positive reaction occurred, the test was terminated within the 45 min.

### 2.8. Statistics

In this study, patient data were entered through Excel software and analyzed by IBM SPSS Statistics 25.0. Those measurement data that matched normal distribution were presented as mean ± standard deviation; otherwise, as a median and interquartile range, frequency and percentage (%) were used for enumeration data.

Spearman correlation analysis: The purpose of this method was to ascertain whether all clinical data variables are correlated with each other and whether each clinical data variable and SS are correlated by calculating the rank correlation coefficient (r_s_) and *p*-value; the larger the r_s_, the stronger the association of the variable and the SS, and an absolute value of r_s_ between 0.3 and 0.6 was considered a relatively strong correlation, and an absolute value of r_s_ > 0.6 was considered a significant correlation. The clinical data variables with statistically significant differences in correlation with the SS were screened with *p* < 0.05. The results of the correlations between all variables generated a heatmap of the correlation matrix (HMCM), where the color saturation was proportional to the strength of the association between the variables, with warm colors indicating a positive correlation and cold colors indicating a negative correlation.

Multiple linear regression analysis: Among the above variables, those significantly associated with SS (*p* < 0.05) and sexwere used as the independent variables and SS as the dependent variable, which were further entered into multiple linear regression analyses using the forward selected method. Adjusted R^2^ > 0.5 indicated that the multivariate linear regression model had a satisfactory fit. A Durbin–Watson test was used to determine residual independence, with Durbin–Watson values between 0 and 4 suggesting that residual independence was met. Collinearity diagnosis was used to determine whether there was an association between variables. If each variable’s tolerance was greater than 0.1 and the VIF was less than 10, it indicated the absence of collinearity between the variables. According to the clinical significance as well as collinearity analysis, factors independently associated with SS were screened (*p* < 0.05).

Receiver operating characteristic (ROC): The mild and severe groups were, respectively, defined according to SS ≤ P25 and SS ≥ P75, and the continuous variables independently correlated with the SS were included in the ROC analysis, and variables’ indicative value for the symptom severity was investigated by calculating the area under the curve (AUC), and an AUC > 0.7 was regarded as a high indicative value. Boundary values were delineated based on the maximum value of the Jordon index, and the associated factors’ sensitivity and specificity for indicating symptom severity were calculated. 

## 3. Results

### 3.1. Primary Characteristics of the Subjects

Two hundred and ninety-six pediatric and adolescent patients hospitalized with a diagnosis of POTS from January 2012 to May 2024 were recruited. There were 129 (43.6%) males and 167 (56.4%) females with a median age of 12 (10, 13) years and a median SS of 6 (4, 9).

### 3.2. Multiple Spearman Correlation Analysis

**Figure 2:**
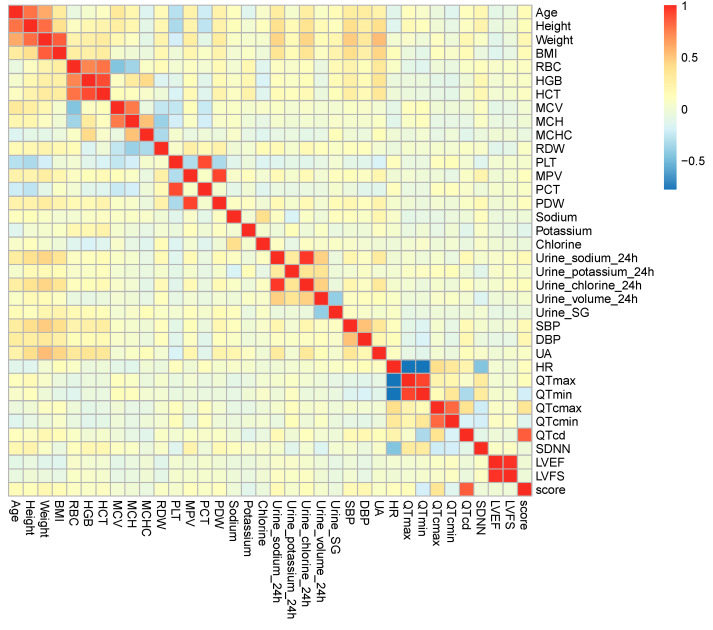
The heatmap of correlation matrix between quantitative variables. The color bar on the right represents the Spearman correlation coefficient, with darker blue indicating a higher negative correlation and darker red indicating a higher positive correlation. BMI, body mass index; RBC, red blood cell count; HGB, hemoglobin; HCT, hematocrit; MCV, mean corpuscular volume; MCH, mean corpuscular hemoglobin; MCHC, mean corpuscular hemoglobin concentration; RDW, red cell distribution width; PLT, platelet count; MPV, mean platelet volume; PCT, platelet hematocrit; PDW, platelet distribution width; Urine SG, urine specific gravity; SBP, systolic blood pressure; DBP, diastolic blood pressure; UA, uric acid; HR, heart rate; QTmax, maximum QT interval; QTmin, minimum QT interval; QTcmax, corrected QTmax; QTcmin, corrected QTmin; QTcd, corrected QT interval dispersion; SDNN, standard deviation of NN intervals in 24 h Holter monitoring; LVEF, left ventricular ejection fraction; LVFS, left ventricular Fraction shortening.

The multiple Spearman correlation analysis was performed to explore the candidate variables correlated with SS. (1) As shown in Table 1 and Figure 2, a total of 13 variables, including age, weight, BMI, serum potassium, 24 h urinary sodium, 24 h urine chlorine, SBP, DBP, QTmax, QTmin, QTcmax, QTcmin, QTcd were correlated with the SS (*p* < 0.05). (2) Among these, age, weight, BMI, 24 h urinary sodium, 24 h urine chlorine, SBP, DBP, QTmax, QTcmax, and QTcd were positively correlated with SS (r_s_ > 0); (3) And serum potassium, QTmin, and QTcmin were negatively correlated with SS (r_s_ < 0). Only QTcd showed a significant correlation with SS (r_s_ > 0.6).

Among the 13 variables associated with SS, significant correlations were determined between age and weight (r_s_ = 0.603), weight and BMI (r_s_ = 0.849), 24 h urinary sodium and 24 h urinary chloride (r_s_ = 0.961), QTmax and QTmin (r_s_ = 0.912), and QTcmax and QTcmin (r_s_ = 0.812), respectively. These correlations all reflected recognized intrinsic connections between the variables.

As far as all quantitative variables were concerned, some recognized intrinsic connections were also revealed. Significant statistical positive correlations were shown between age and height (r_s_ = 0.769), height and weight (r_s_ = 0.789), RBC and HGB (r_s_ = 0.725), RBC and HCT (r_s_ = 0.782), HGB and HCT (r_s_ = 0.903), MCV and MCH (r_s_ = 0.767), PLT and PCT (r_s_ = 0.900), MPV and PDW (r_s_ = 0.918), and LVEF and LVFS (r_s_ = 0.978), respectively; variables with a significant negative correlation were found between HR and QTmax (r_s_ = −0.781) as well as HR and QTmin (r_s_ = −0.764), all *p* < 0.001 (Figure 2).

### 3.3. Multiple Linear Regression Analysis of Factors Relevant to SS

#### 3.3.1. Collinearity Statistics Among Variables

As intrinsic connections between the paired variables were found among the 13 variables statistically related to SS, collinearity analysis was performed before the regression analysis. The results showed that among the 13 variables above, 7 variables, including weight, 24 h urinary sodium, 24 h urinary chlorine, QTmax, QTmin, QTcmax, and QTcd, had collinearity (tolerance < 0.1, VIF > 10) with each other, as shown in Table 2.

#### 3.3.2. Excluding Variables That Show Collinearity

Combined with the clinical significance, 24 h urinary sodium, QTmax, and QTcd variables were retained, collinearity statistics were performed again, and there was no collinearity between the quantitative variables (tolerance > 0.1, VIF < 10), as shown in Table 3.

#### 3.3.3. Multiple Linear Regression Analysis

Sex and quantitative variables were investigated in the multivariate linear regression analysis, and eventually, QTcd alone (β = 0.839, *p* < 0.001) was independently correlated with SS, as shown in Table 4.

#### 3.3.4. Obtaining the Regression Equation

The regression equation was SS = −4.482 + 0.242 × QTcd. (F = 698.300, *p* < 0.001, adjusted R^2^ = 0.703, suggesting that the regression equation was significant and the multiple linear regression model fit well). For every 1 ms increase in QTcd, the SS was increased by 0.242 points.

### 3.4. ROC Analysis of QTcd Indicating the Symptom Severity in Children with POTS

In order to test the efficiency of QTcd for indicating the symptom severity of pediatric POTS, the mild group (SS ≤ P25), including 110 patients, and the severe group (SS ≥ P75), including 84 patients, were defined based on the SS. The basic data of the two groups are shown in Table 5. The efficiency of QTcd for distinguishing the two groups was assessed by the ROC curve.

**Figure 3:**
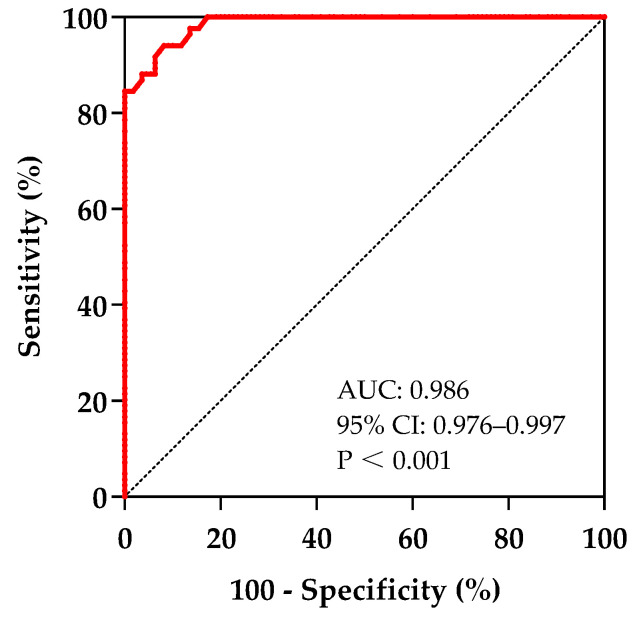
The ROC curve of QTcd indicates the symptom severity in children with POTS. The sensitivity and specificity for symptom severity indication were 94.0% and 91.8%, respectively.

The results of the ROC analysis showed that the AUC of QTcd for indicating the mild or severe group was 0.986 (95% CI 0.976–0.997), which indicates high distinguishing efficiency (Figure 3).

When the Jordon index was at its maximum, the cut-off value for QTcd was 45 ms. Thus, taking QTcd > 45 ms as a condition to indicate a severe case of POTS in children and adolescents, the sensitivity was 94.0%, and the specificity was 91.8%.

## 4. Discussion

POTS is often manifested by a chronic condition that presents with multisystem symptoms in addition to the persistent tachycardia that occurs under a long-standing state. Children’s daily lives are seriously threatened by these frequent symptoms of orthostatic intolerance, which meanwhile bring a huge psychological burden on them. Exploring biomarkers for indicating the condition using simple and readily available routine clinical data would be beneficial in optimizing and guiding the management of pediatric patients with POTS. The current research revealed that QTcd was positively correlated with the SS of children with POTS, and the established multiple linear regression model had high discriminative properties. The value of QTcd in indicating symptom severity was acceptable (AUC = 0.986), and the sensitivity and specificity of discriminating symptom severity were high when a cut-off value of 45 ms was used, which was 94.0% and 91.8%, respectively. That is, a patient with QTcd > 45 ms may be more likely to exhibit serious clinical manifestations, indicating that the patients should be paid more attention to and closely followed up to achieve more optimized management. In addition, multiple correlation analysis showed that, among the 13 factors related to SS, no significant correlations between each other were found except for some recognized intrinsic connections.

Increased sympathetic excitability during the orthostatic posture is one of the most important characteristics of POTS [20]. As a normal circumstance, venous return, cardiac filling, and cardiac output are decreased during the upright contrasted with the lying position [34], and the stimuli perceived by the pressure receptors are consequently weakened, which compensatively elicits sympathetic activation to maintain heart rate and blood pressure [35,36,37,38]. The decreased blood return can be exacerbated when there is insufficient circulating blood volume and impaired peripheral vasoconstriction, both of which can cause excessive activation of sympathetic function [20,21,39]. In addition, many patients with POTS also have a primary hyperadrenergic state, and the sympathetic tone is markedly increased, and even more when standing [40,41], which can cause a marked increase in heart rate [42], and as a result, their discomfort is more pronounced when they are upright.

In this study, we revealed a strong positive correlation between QTcd and symptom severity in children with POTS. Specifically, children with larger QTcd may suffer from more severe symptoms. The QT interval refers to the duration of ventricular depolarization and repolarization, which is affected by the states of sympathetic and parasympathetic nerve activity. QT interval dispersion (QTd) is obtained by subtracting QTmin from QTmax in the electrocardiogram, reflecting the inhomogeneity of ventricular myocardial depolarization and repolarization process [29,43,44,45]. The sympathetic nerves are unevenly distributed within the heart [46,47], and generally, the inhomogeneity of ventricular depolarization increases when sympathetic tone increases. In addition, QTd also depends on the difference in the duration of myocardial repolarization [48], which leads to delayed ventricular repolarization when parasympathetic activity decreases. In short, both increased sympathetic and decreased parasympathetic activity exacerbate the inhomogeneity of ventricular depolarization and repolarization, resulting in an enhanced QTd. QTcd is the heart rate-corrected QTd, and thus, QTd and QTcd are intrinsically determined by the inhomogeneity and electrical instability of the impaired autonomic nervous system within the myocardium [30,49,50,51]. Previous studies have shown that most patients with POTS are characterized by increased sympathetic and decreased parasympathetic activity, especially during standing [52,53], which is directly related to orthostatic tachycardia as well as symptoms of orthostatic intolerance [53]. Therefore, we speculated that, while QTcd can well assess the changes in autonomic functioning [49], among the children with POTS, those with greater QTcd may have more excited sympathetic activity, which may account for the more severe symptoms.

Previous studies have also been conducted on the indicators for severity of symptoms in children with POTS [17,18,19]; however, in these studies, biomarkers related to the condition of children with POTS, such as plasma Hcy, sulfur dioxide, and serum resistin, were not routinely detected, especially in the grassroots medical institutions. In the present study, we collected the common clinical indices that are closely related to the mechanisms of POTS (as mentioned above), and the indicators were more convenient to obtain.

We reported the indicators of the condition of children with POTS in this study. We found that QTcd exhibits good sensitivity and specificity in indicating the severity of symptoms among pediatric and adolescent patients with POTS, which will facilitate the assessment of the condition and disease management. Patients with potentially severe symptoms should be given more active disease monitoring and treatment. Meanwhile, QTcd obtained by 12-lead ECG has its own advantages in clinical practice. The acquisition of QTcd is simple, non-invasive, and inexpensive. There is no need for blood specimen collection or 24 h urine collection as many other indices. The measurement can be performed by a licensed physician, which is conducive to its application and promotion in various medical institutions.

However, the current research contains its own limitations. The participants of the research were from a single center, the sample size was small, and the retrospective nature may have some bias. For example, the measurements of some variables may lack quality control. The participants in the current study were single-center, hospitalized patients who may have more severe symptoms than the outpatient and those who did not visit the doctors, which may result in selection bias, and its generalizability will be limited to some extent. Further prospective studies could be conducted to create symptom-scoring forms and count and calculate symptom scores for each patient eligible for enrollment in the study at the time of admission to minimize recall bias. In addition, multi-center cohort studies involving both outpatient and inpatient children are needed for further validation and to expand the sample size and the source of participants to reduce the effect of bias.

## 5. Conclusions

In conclusion, the current research suggested that QTcd was positively correlated with symptom severity in pediatric cases with POTS and had a high indicative value for symptom severity. Our findings may help indicate potentially severe cases in pediatric patients with POTS, which will facilitate stratified management of the disease and more aggressive treatment of severe patients.

## Figures and Tables

**Table 1:** Spearman correlation analysis of clinical data variables with symptom score.

Variables		r_s_	*p* Value
Baseline data			
Age		0.133	0.022
Height		0.112	0.055
Weight		0.172	0.003
BMI		0.130	0.025
Variables related to blood volume			
Blood cell count	RBC	0.087	0.134
	HGB	0.064	0.274
	HCT	0.082	0.160
	MCV	0.015	0.797
	MCH	−0.015	0.797
	MCHC	−0.063	0.282
	RDW	0.065	0.263
	PLT	0.030	0.603
	MPV	0.102	0.081
	PCT	0.062	0.286
	PDW	0.109	0.060
Serum ion	Sodium	−0.030	0.605
	Potassium	−0.117	0.045
	Chlorine	−0.038	0.510
24 h urine	24 h urinary sodium	0.148	0.011
	24 h urinary potassium	0.031	0.595
	24 h urinary chlorine	0.117	0.045
	24 h urinary volume	0.025	0.673
Urine SG		−0.045	0.445
Variables related to vascular function			
SBP		0.155	0.008
DBP		0.167	0.004
UA		0.073	0.210
Variables related to autonomic nervous function			
12-lead ECG	HR	0.096	0.100
	QTmax	0.116	0.046
	QTmin	−0.195	0.001
	QTcmax	0.343	<0.001
	QTcmin	−0.136	0.019
	QTcd	0.849	<0.001
24 h Holter	SDNN	−0.018	0.754
Echocardiography	LVEF	−0.006	0.919
	LVFS	−0.019	0.748

r_s_, rank correlation coefficient; BMI, body mass index; HGB, hemoglobin; RBC, red blood cell count; HCT, hematocrit; MCV, mean corpuscular volume; MCH, mean corpuscular hemoglobin; MCHC, mean corpuscular hemoglobin concentration; RDW, red cell distribution width; PLT, platelet count; MPV, mean platelet volume; PCT, platelet hematocrit; PDW, platelet distribution width; Urine SG, urine specific gravity; UA, uric acid; SBP, systolic blood pressure; DBP, diastolic blood pressure; 12-lead ECG, 12-lead electrocardiogram; HR, heart rate; QTmax, maximum QT interval; QTmin, minimum QT interval; QTcmax, corrected QTmax; QTcmin, corrected QTmin; QTcd, corrected QT interval dispersion; SDNN, standard deviation of NN intervals in 24 h Holter monitoring; LVEF, left ventricular ejection fraction; LVFS, left ventricular Fraction shortening. A statistical difference is shown by a *p* value < 0.05.

**Table 2:** Analysis of collinearity between age, weight, BMI, serum potassium, 24 h urinary sodium, 24 h urine chlorine, SBP, DBP, QTmax, QTmin, QTcmax, QTcmin, and QTcd.

Variables	Collinearity Statistics	
	Tolerance	VIF
Age	0.373	2.684
Weight	0.100	10.019
BMI	0.167	5.985
Serum potassium	0.913	1.095
24 h urinary sodium	0.070	14.278
24 h urinary chlorine	0.072	13.947
SBP	0.548	1.825
DBP	0.680	1.470
QTmax	0.001	914.549
QTmin	0.001	1058.700
QTcmax	<0.001	-
QTcmin	0.151	6.626
QTcd	0.007	141.591

VIF, variance inflation factor; BMI, body mass index; SBP, systolic blood pressure; DBP, diastolic blood pressure; QTmax, maximum QT interval; QTmin, minimum QT interval; QTcmax, corrected QTmax; QTcmin, corrected QTmin; QTcd, corrected QT interval dispersion.

**Table 3:** Analysis of collinearity between age, BMI, serum potassium, 24 h urinary sodium, SBP, DBP, QTmax, QTcmin, and QTcd.

Variables	Collinearity Statistics	
	Tolerance	VIF
Age	0.777	1.286
BMI	0.709	1.410
Serum potassium	0.963	1.038
24 h urine sodium	0.814	1.229
SBP	0.631	1.585
DBP	0.690	1.449
QTmax	0.884	1.131
QTcmin	0.868	1.152
QTcd	0.856	1.168

VIF, variance inflation factor; BMI, body mass index; SBP, systolic blood pressure; DBP, diastolic blood pressure; QTmax, maximum QT interval; QTcmin, corrected QTmin; QTcd, corrected QT interval dispersion.

**Table 4:** Multiple linear regression analysis of factors relevant to symptom score (n = 296).

	Unstandardized Coefficient		Standardized Coefficient			Collinearity Statistics	
	B	SE	β	t Value	*p* Value	Tolerance	VIF
Constant	−4.482	0.436		−10.272	<0.001		
QTcd	0.242	0.009	0.839	26.425	<0.001	1.000	1.000
F value 698.300; Adjusted R^2^ 0.703; Durbin–Watson 2.038							

SE, standard error; VIF, variance inflation factor; QTcd, corrected QT interval dispersion.

**Table 5:** Primary characteristics of patients in the mild and severe group.

	Mild Group	Severe Group
SS (scores)	3 (2, 4)	11 (9, 12)
QTcd (ms)	34.42 (30.98, 39.91)	59.51 (52.04, 66.52)
Age (yrs)	11 (10, 13)	12 (11, 14)
Male [n(%)]	55 (50.0%)	34 (40.5%)
Female [n(%)]	55 (50.0%)	50 (59.5%)

SS, symptom score; QTcd, corrected QT interval dispersion.

## Data Availability

The original contributions presented in this study are included in the article. Further inquiries can be directed to the corresponding author.
